# Co-creation of educational commons spaces to reverse inequalities: project SMOOTH and the Children's Club

**DOI:** 10.3389/fsoc.2023.1235782

**Published:** 2023-08-15

**Authors:** Natália Fernandes, Teresa Sarmento, Marlene Barra, Daniela Silva, Fernanda Martins, Joana R. Casanova

**Affiliations:** ^1^Research Centre on Child Studies, Institute of Education, University of Minho, Braga, Portugal; ^2^Research Centre on Education, Institute of Education, University of Minho, Braga, Portugal

**Keywords:** educational commons, children, social integration, democratic citizenship, inequalities, action research

## Abstract

This article presents an action-research project from the EU-funded SMOOTH project, which focuses on the potential of Educational Commons to address educational inequalities. The project adopts an emergent paradigm that views spaces for collaboration, content co-creation, socialization, governance, and play as catalysts for reversing inequalities. The action-research, conducted in a disadvantaged non-formal education setting in northern Portugal, involved children aged 8–10 years old. Over a span of 10 months, the innovative action-research program aimed to achieve several objectives: (1) reversing inequalities faced by vulnerable social groups, (2) strengthening inter-cultural and inter-generational dialogue and social integration, (3) developing essential social and personal skills, and (4) creating smooth spaces of democratic citizenship based on equality, collaboration, sharing, and caring. By understanding the tensions and conflicts that emerge in children's everyday situations, the project sought to build and foster community through embracing differences. This article analyzes the characteristics, behaviors, challenges, and strengths observed during the 30 sessions. The results provide insights into the dimensions of Children as commoners, in terms of sharing and care, cooperation and collective creativity and active citizenship. This research contributes to the exploration of Educational Commons as a means to promote equity and transform educational contexts.

## Introduction

This article reflects on the data resulting from an action research project-The Children's Club-developed within the scope of the European project SMOOTH: “Educational Commons and Active Social Inclusion.” The SMOOTH project is funded by the Horizon 2020 program and led by the University of Thessaloniki (Greece), involving a consortium of universities and organizations from Spain, Italy, Sweden, Belgium, Germany, Estonia and Portugal.

The SMOOTH project (2021–2024), involves the participation of approximately 50 educational institutions (formal and non-formal education) where research projects have been carried out with the aim of understanding, developing, and accelerating the potential impact of common goods in Education for the reduction of inequalities and the promotion of active social inclusion of children and youth.

The SMOOTH is based on the conception of the common good, in which education is a powerful tool for its achievement and as a common good in itself. When referring to common goods in education, we are talking about knowledge and educational opportunities accessible to all, regardless of socio-economic conditions and geographical location. It is crucial to break down the barriers traditionally faced in accessing education in order to promote a more equitable, democratic, and inclusive society where everyone has the opportunity to fully develop their potential.

To implement these assumptions, the Portuguese team, from the University of Minho (hereinafter referred to as Smooth-UMinho), in collaboration with a partner, Porta 7, developed the Children's Club, one of the action research projects that will be presented throughout this text.

We will present two interconnected parts. In the first part, we present some theoretical approaches to the concept of education as a common good and children as commoners, emphasizing the importance of conceptualizing children as social actors in the operationalization of the dimensions encompassed by SMOOTH. The second part analyzes the Children's Club project and the way how children as commoners can be considered. The final section synthesizes the reflection arising from the entire process.

## Definition of the common good

The common good is a polysemic term that has acquired distinct meanings, especially in recent decades. The concept was appropriated in the 1980s by neoliberal economics in discourses promoting globalization as a common good (Kostakis and Bauwens, 2019). However, it was soon realized that this common good was primarily associated with a business and mercantilist connotation, linked to profit and the exploitation of labor. Challenging this conception, authors such as Pato et al. (2013) and Korsgaard (2019) advocate common goods are a living and active process that materializes through acts of mutual support, conflict resolution, communication, and experimentation to create systems for managing shared resources. This approach challenges the values of neoliberalism, which emphasize individualization, competition, and disengagement from political and collective life (Pechtelidis and Kioupkiolis, 2020). Instead, it promotes values of equality, openness, sustainability, and ecological attitudes and behaviors. The commons represent a transformative vision aimed at improving everyday life, presenting an opportunity to reimagine our collective future and revolutionize social organization, economics, infrastructure, politics, and state power. As a social form, the commons enable individuals to experience freedom without suppressing others, establish fairness without bureaucratic control, foster a sense of togetherness without coercion, and assert sovereignty without promoting nationalism.

The notion of the common good also emerges in relation to goods available to all members of a community, in contrast to individual interests. However, it is not solely “the harmonious life of individuals that matters, but also the harmony of the life that human beings have in common” (UNESCO, 2015; p. 85). In this sense, the harmonious life that human beings have in common need to consider resources and conditions that belong to everyone and from which society as a whole should benefit, that includes both natural resources and cultural and historical assets. It is within this context that it is important to reflect on the role of education as a common good.

###  Education as a common good

The concept of education as a common good need, above all, to be aware about the meaning of “good” and “common”.

To speak of “good” implies discussing a value, and according to Casa-Nova (2021), values are choices individuals make based on their personal and social needs and references. Thus, human values constitute a central dimension in social life, and the existence of a society without a minimum set of common values is not possible. Halsted argues that these values should include a basic social morality, the acceptance of a common system of law and government by all societal groups, and a commitment to values presupposed by the pluralist ideal (1996, p. 15).

Values serve as references for thought and action, guiding social relationships, moral behavior, the sense of belonging to social groups, and ways of being and acting.

Common goods, as social values, demand, according to Restrepo, “the presence of more dialogue and paths through which values permeate, opening up space for interpersonal experiences” (2004, p. 65). From the perspective we endorse, the intensification of informal and playful dialogues can establish an empathetic network within society—a network characterized by greater tolerance, harmony, trust, cordiality, self-confidence, and openness to others. In this context, a key idea to emphasize is that education as a common good should be conceived in a way that meets the needs of all individuals and implies a set of shared goals and values, such as promoting equal opportunities for all, regardless of socioeconomic background, race, gender, and other factors. It involves providing individuals with essential skills and knowledge for active citizenship, enabling them to contribute to the well-being of the community.

Referring to the emphasis placed by the Organization for Economic Cooperation and Development on “knowing how” and “using” within the framework of developing skills for the future (OECD, 2018), we can add that active participation is fundamental for the development of skills and attitudes that enable teamwork, communication, conflict resolution, as well as the development of critical awareness to address social and environmental issues, thereby improving society and making it environmentally sustainable.

Thus, education based on common goods is seen as an alternative system of action, with values based on equality and peer governance. As proposed by the SMOOTH project, it involves community-based horizontal and democratic approaches that contribute to the reversal of inequalities. The concept of reversing inequalities is founded on the principle that every individual, regardless of age, possesses the right and knowledge to participate in decision-making processes regarding significant aspects of their lives. This belief is instrumental in empowering children and promoting their active engagement in public life. By enabling children to have a voice and be involved in democratic processes, we contribute to their well-being, social inclusion, and the transformation of society toward a more democratic and equitable future (Pechtelidis and Kioupkiolis, 2020).

Creating spaces that are designed and operated by and for children becomes a possibility when democratic values, mutual respect, autonomy, and solidarity are embraced and fostered. These values serve as a foundation for nurturing an environment where children's voices are valued and their agency is recognized, leading to their active participation in shaping their own lives and the communities they belong to. In spaces where children actively participate for the common good, peer governance is emphasized minimizing hierarchies and promoting collaboration, shared responsibility, and mutual respect. By embracing diversity, consent in decision-making, and generous knowledge sharing, it provides a protected space for commoning, challenging traditional notions of ownership and control to address inequality and foster inclusivity.

In this sense, this process involves a reconfiguration of education based on the values of participation, sharing, and care by all parties of the educational community, with children, teachers, and parents co-participating. Children's participation will enhance agency (Korsgaard, 2019). This can be achieved when the effective exercise of children's rights, enshrined in the United Nations Convention on the Rights of the Child (UNCRC, 1989), is promoted.

Above all, the political rights that emerge from the UNCRC (1989), particularly from articles such as Article 12, Article 13, Article 14, and Article 17, provides strong support for sustaining new subjectivities in the public arena—the subjectivities of children—recognizing their social action with significant influence in organizing public life, as stated by Pechtelidis and Kioupkiolis (2020):

“*(...) both within the formal political system and beyond it, at micro, meso, or macro levels of social life, in more or less institutionalized social spaces and relationships, visible in any social field, including those that oppose or go beyond the political system. Based on this idea of the political, we explore the collective action of children (and adults) in social relations and subjectivities in their everyday activities” (p. 2)*.

## The child as a “commoner”

The collective construction of the common good, being intrinsically dependent on participation, leads us to the importance of conceptualizing children as social actors and active subjects of rights (Fernandes, 2009, 2019; Fernandes and Trevisan, 2018).

The civic status of children has been evolving, recognizing them as social actors who are competent to make choices and express ideas, with their own agenda (Sarmento, 2005, 2008, 2013, 2016). Ideally, their education should enable them to become “more capable of sustaining processes of authorship and autonomy” (Demo, 2003, p. 53). The participation of children in processes initially directed toward them implies recognizing their “capacity to think and act upon themselves” (Dahlberg et al., 2003; p. 162), as well as others, children or adults, and situations and ideas. From this perspective, “institutions dedicated to childhood should be seen as the social construction of a community of human agents, originating from our active interaction with other people and society” (Dahlberg et al., 2003, p. 87).

The sociology of childhood, in particular, contributes significantly to the social concern for children in a global context, deeply marked by conflicts and contradictions, that place children at the center of a set of paradoxes (Qvortrup, 1991; Pinto and Sarmento, 1997). Sociology of childhood questions the social constructions of childhood in different contexts, the structural condition to which children belong and by which they are objects of conceptualizations and interpretations of ways of being and acting (Alanen, 2011). Within these reflections and breaking away from classical approaches to socialization, that viewed children as passive beings in the educational process, a new perception of the child emerges, one that we subscribe to: the child as a social actor (Marchi, 2017; Baraldi and Cockburn, 2018; Pechtelidis, 2021), or the child-citizen, a subject with rights, particularly the right to democratic participation, including decision-making (Fernandes, 2009, 2019; Fernandes and Trevisan, 2018).

The sociology of childhood helps to disseminate Childhood as a social category and the child as an actor, considering that this conception (Sarmento, 2013; p. 35):

“*(…) is by no means a prejudice or a politically generated idea: it is a long process of rescue, similar to those carried out with subaltern categories (women, blacks, post-colonial peoples, homosexuals) that classical science has traditionally underestimated and ignored.”*

The affirmation of the child as a social actor prioritizes the analysis of their actions as active and creative agents, who transform and reproduce reality in diverse ways, negotiating with adults and their peers, and developing new communicative, linguistic, discursive, and action-oriented forms that are added to the dominant adult culture (Buckingham, 2002). The analysis and unraveling of these processes of interpretive reproduction (Corsaro, 2002, 2017) within the peer culture is one of the challenges of the Sociology of Childhood, and the centrality of the action and voice of social actors as partners in research is also a characteristic of social studies of Childhood.

Equally important is questioning how the right of children to participate and express their opinions and actions in the public arena is presented. Pechtelidis and Kioupkiolis (2020, p.2) argue that considering children's participation in common processes involves political and practical dimensions of their agency, their possibilities of influencing the life contexts in which they move, which implies respect for their voices, but also respect for their choices.

Therefore, we argue that children's participation is a fundamental axis in educational contexts based on and promoting the common good. However, for this common good to effectively occur, it is necessary to mobilize an epistemological vigilance regarding how it can be put at the service of collective processes of common good construction. These are processes in which children feel that they are part of, contributing to the construction of logics of care, freedom, solidarity, and democracy, which can only happen if children are actively involved in more inclusive and participatory dynamics, in collective and intergenerational dynamics of co-constructing knowledge and shared management.

## The Children's Club and the construction of a common space

### Presentation and discussion of data

Designing educational practices and relationships that are based on the principle of Education as a common good critically involves mobilizing collective freedom and experimentation, participation, solidarity, care, and sharing, bringing new possibilities for a balanced exercise of power.

In the Children's Club, based on the insights of Alderson and Montgomery (1996), Fernandes and Tomás (2011), and Fernandes and Trevisan (2018), we aimed to ensure that children engage in decision-making processes. For this purpose, it was essential to work with them on dimensions related to their right to be informed, to express informed opinions, and to organize themselves in spaces that ensure their opinions are considered, so they feel part of the decision-making process. Thus, based on an understanding of children as active rights-holders, the Children's Club was constructed as a physical and symbolic space, to facilitate shared governance in the co-construction of situated knowledge that is meaningful for both children and adults and promotes equity.

###  Some methodological notes on the Children's Club project

The Children's Club emerged from a partnership between teachers, professionals from the hosting institution, and the Smooth-UMinho team, with the aim of gaining a deeper understanding of the group of children who would be involved in it. The third party has been actively involved in the community for the past 15 years, recognizing the challenges faced by children and their families. These challenges include a lack of opportunities for democratic participation in decision-making processes, difficulties in accessing essential services such as education, healthcare, and employment, and the perpetuation of mechanisms that contribute to social exclusion. The teachers and professionals from the third party emphasized the importance of allowing children to share their voices and interests, understanding the pressures imposed by their daily routines. The initial interactions between the Smooth-UMinho team, children, and professionals from the third party took place in a non-formal setting within a primary school, as part of academic activities. The team invited the children to join and collaborate in decision-making processes, emphasizing that their participation or presence was optional and that non-participation would not result in any prejudice. The choice to participate was left to each child, respecting their individual decisions during each contact. Children showed immediate curiosity and interest in the project, and after getting to know each other, the children actively participated in defining the project's goals, and expressed their desire to create “a space in which all decisions and activities will be made by everyone: to talk, to play, to sing, to do all the things related to theirs interests.”

The research-action project was developed between March 2022 and January 2023, with the first phase taking place from March to July 2022, followed by a pause during which the team reflected on the process to prepare for the second phase, which occurred from September 2022 to January 2023.

The project was driven by the following main goals:

- To challenge the prevailing narratives regarding the role of education, inclusion, and childhood.
- To foster shared governance, allowing children and adults to have reduced governance and enabling new opportunities for a balanced exercise of power.
- To facilitate learning through co-construction, emphasizing the importance of local and emancipatory knowledge.

The weekly dynamics involved 1 day of preparation before entering the field, 2 days of implementing the pedagogical scenario, and 1 day for reflection and organization of data.

The project team based their work on qualitative methodology, using the research-action method (Carr and Kemmis, [1986], 2012; Descombe, 1999). Activities promoted active listening and dialogue with the children, focusing on topics related to their rights and well-being, in order to promote social inclusion, foster their active citizenship and reversing inequalities. It was employed a variety of data collection techniques to ensure that the perspectives of the children were heard and taken into consideration during the decision-making process, resulting in the creation of a space that was collectively conceived and designed. The team prioritized a participatory approach, actively encouraging the involvement of the children in the conceptualization and organization of the Children's Club, a space created with, by, and for the children, with the purpose of developing activities relevant to their interests and needs, as well as promoting social inclusion and active citizenship.

Data was generated through a variety of techniques, including participant observation, interviews, field notes, and analysis of documents produced during the sessions, such as photographs and video recordings. The action and data collection methodologies had to be adapted and refined during the research process to accommodate the unique characteristics of the children involved.

The group was restless, characterized by interpersonal conflicts and frequent incidents of verbal and physical aggression. As a result, the research team initially had to closely guide the process to support the development of self-regulation skills among the children (Ferrés and Masanet, 2017) enabling them to effectively express their opinions and make collaborative decisions. Researchers and children were involved in data generation, while the final interpretation was conducted exclusively by the research team.

The analysis was carried out based on a qualitative content analysis process with triangulation of the data collected from different techniques employed following the organization of data according to a categorical framework constructed based on the dimensions indicated below.

## Results

###  Common practices between children and adults in the Children's Club sharing and care

In the UNESCO report (2020) it is advocated that “this new contract should be based on pedagogies of cooperation and solidarity. These pedagogies should be grounded in principles of non-discrimination, respect for diversity, and restorative justice, as well as being conceived from an ethic of care and reciprocity” (p. 48).

Cooperation and solidarity, at their core, refer to the concepts and, above all, to the social practices of care and sharing. As Borges-Duarte (2019) states, care constitutes a central element of being human; it is through care that we can understand the most remarkable characteristics of humanity: its exposure to the surrounding world and its expression through openness and responsiveness to what surrounds it. In this sense, care extends to sharing, as it originates from each individual's care for themselves and extends to the Other with whom they live, becoming a common good. When children engage in sharing and caring behaviors, they develop a sense of unity and collective responsibility toward each other. By promoting these practices, the Children's Club creates an environment where children learn to work together, support each other, and recognize the importance of equitable distribution.

Through observation, several situations demonstrating different attitudes were recorded, as evidenced by subsequent field notes:

*In a moment intended for a shared meal, Child C19 says*,

“*Get out of here! This snack is ours.”*

*Two children (Child C6, and Child C7) immediately state that there is enough for everyone, that it is too much for one person (Field note, 12/13/2022)*.

Child C6 takes several cookies on a napkin and looks for the educational assistant to offer one. The remaining cookies are wrapped in a napkin and stored in the backpack to take home (Field note, 07/05/2022).

The data show us that the children in the group had notable difficulty in sharing and taking care of their peers. However, it is also clear that they displayed caring attitudes toward those closest to them. Sharing and care are rooted in the concept of the common good, where individuals consider the well-being of others as essential. Through sharing resources, such as food or materials, children learn to prioritize the needs of the group over their individual interests. This cultivates a sense of empathy and fosters a community spirit within the Children's Club.

Another example is when Child C4, with his hand raised to physically assault a peer, stops for a second and refers to one of the adults, saying, “I will use my words, I will use my words!” He then retreats, lowers his hand, and demonstrates emotional control, showing concern for his peer. This example highlights the potential for growth and development in empathy and emotional control. By providing a supportive environment, the Children's Club helps children recognize the importance of using their words instead of resorting to physical aggression, demonstrating concern for the well-being of their peers. Additionally, in some musical activities that were conducted, a single instrument was presented to the class as a form of collaborative work and care. Through these strategies, children learn to collaborate, respect each other's contributions, and collectively create something meaningful. This promotes a culture of collaboration and care within the group, contributing to the reversal of inequalities by emphasizing the value of collective efforts.

#### Cooperation and collective creativity

The ability of children and adults to engage in joint dynamics, sharing common goals related to their own transformation and the transformation of broader contexts, is one of the defining aspects of sustained educational processes that promote the common good, allowing the development of a sense of collective purpose and a shared commitment to addressing inequalities.

In the Children's Club, dimensions of cooperation and collective creativity gradually emerged as the project developed. As mentioned earlier, the children initially had difficulties engaging in cooperative processes. The intervention of adults intentionally created opportunities for the development of these skills, starting with the establishment of a space that was already familiar to them, with a well-maintained and welcoming physical appearance, indicating care for that group of children, in order to face a pattern of relationships characterized by physical and verbal violence and without space for the Other.

The activity described below-the composition of a music piece known as the Children's Club Music - became central to creating renewed social practices among the children and with the adults.

This process unfolded over 8 sessions during the second phase of the project. It began with the children's involvement in musical activities, during which they demonstrated interest and commitment, making it clear to the adults that music could be a powerful language to engage their interest and participation, as well as fostering their creativity.

**Figure 1:**
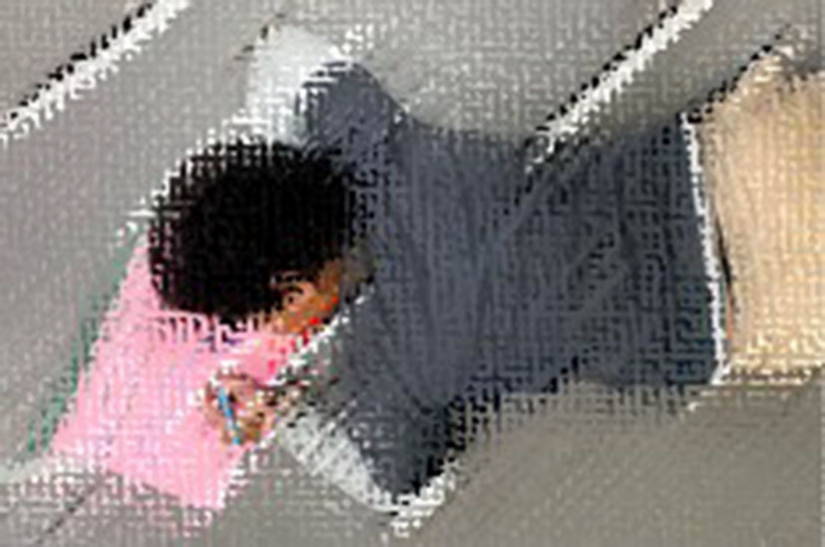
Child writing ideas to include in the lyrics.

This task started with initial conversations with the children, during which it was noticed that activities involving rhythms captured their attention. This led the adults to inquire about the type of music they liked and challenged them to create a piece that represented the group. Thus, through conversation, the children started collectively defining the main theme of the music. They then explored and worked in small groups to identify key ideas and collaboratively create lyrics, ensuring that all group members agreed with the different proposals (see Figure 1). Working in small groups facilitates the children's participation and interaction in the creative processes, which are later shared with the larger group. By involving children in the collective decision-making process, they develop a sense of ownership and agency, contributing to the reversal of inequalities by valuing their perspectives and contributions. With the diversity of contributions, the adults collaborated with the children to compose the final version of the music.

The music was seen as a social practice and a language capable of engaging the children's interest and participation, as well as fostering their creativity through the sharing and discussion of different proposals. Several dimensions of cooperation and collective creativity can be identified in the activity of composing the Children's Club Music. Working in small groups was a strategy that facilitated the children's participation and interaction in the creative processes, later shared with the larger group.

This activity allowed for the creative expression of the children, both individually and collectively, promoting social interaction. The composition of the Children's Club Music becomes a central activity that fosters cooperation and collective creativity. The children were able to work in small and large groups, sharing ideas, communication skills, and active listening. All children had equal opportunities to participate and collectively make decisions about the lyrics and music. This activity promotes collaboration, shared responsibility, mutual respect, and cooperative decision-making processes.

It was significant to observe how the children enthusiastically and spontaneously engaged in musical and choreographic activities, which can be interpreted as a demonstration of collective cooperation, creativity, and a sense of community identity. The enthusiastic and spontaneous engagement of children in musical and choreographic activities reflects a sense of collective cooperation, creativity, and a strong community identity, creating a sense of belonging and fostering a supportive and inclusive environment within the Children's Club.

### Active citizenship

Participation and active citizenship are inseparable dimensions of educational processes that promote the common good. While citizenship refers to the exercise of social and political rights by citizens, it is important to note that it does not inherently guarantee principles such as equality, justice, and dignity for adult citizens, let alone child citizens. This is because, in general social contexts and many educational contexts in particular, children are often viewed as not-yet-citizens, justified by arguments that portray children as incompetent, inexperienced, not yet mature, and unprepared to participate in public life, as advocated by Prout (2005).

Too often, children are expected to fit into adult modes of participation when what is needed is institutional and organizational change that encourages and facilitates children's voices. Unfortunately, children's participation is often high in rhetoric but low in practice, as stated by Prout (2005).

We consider childhood citizenship to involve the full recognition of children not as recipients, passive receivers, consumers, or even beneficiaries of society, but as active, participatory, and co-responsible subjects in their own lives (Tomás and Fernandes, 2013; Fernandes and Trevisan, 2018). To meet this image of the child, the institutions where children are involved-such as daycares, schools, leisure workshops, clubs, care homes, and foster homes-must provide spaces for children's autonomous expression. As advocated by Griffith (1998), spaces for children's participation should provide opportunities for them to be critically reflective, morally autonomous, and socially active. By creating spaces that encourage children's autonomous expression and participation, institutions like the Children's Club empower children to actively engage in decision-making processes and contribute to the development of common goods. Emphasizing the aforementioned Children's Rights (Articles 12–15, and 17 of the UNCRC, 1989), we can succinctly demonstrate how these rights intertwine in the process of developing common goods in the daily life of the Children's Club.

The dimension of care is linked to the dimension of participation (Article 12) whenever the space of the Children's Club is used to develop activities that children and adults consider relevant (Article 13) and/or to address their own needs (Article 14). Cooperation (Article 15) is highlighted in the context of the participation dimension that underlies it when children (re)recognize and establish favorite places within the constructed space and when there is the possibility of interaction between the children and the adults of the SMOOTH team.

Finally, the dimension of participation and sharing is emphasized when the space of the Children's Club provides access to information (Article 17) that can support the overcoming of identified problems and promote social inclusion and active citizenship.

Since the beginning of the intervention, the children have shown difficulties in understanding and following established rules, often invading each other's space and interrupting one another, although some demonstrated the ability to regulate rules among their peers. As a result of the need to structure the group and avoid adults always regulating relationships within the group, negotiations were promoted with the children to establish the rights and responsibilities of each individual, demonstrating collaborative efforts to establish a healthy environment for coexistence.

The following excerpt illustrates a process of collective negotiation to define rights and responsibilities within the group:

*Child C4, in the previous phase (1st phase of the project), they say, “We participated in all that noise.” W5 asked what they should do this year to reduce the noise. (...), W1 asks, “What should we do to avoid having to shout all the time inside the room?”*

*W5 suggests, “Can I suggest something? Let's think of rules to make the Children's Club a quieter and more peaceful place. Everyone can think and then share and write a rule on the board. What do you think?” The children agree and start recording their proposals. In this process, there is a lot of commotion, no one is interested in listening to their peers' suggestions, and some children start crying because they are being hurt by their peers. W5 gathers the group to discuss this problem and think about how they can collectively do something to change this situation*.

“*Do you have any suggestions, Child C4?” W5 asks Child C4, one of the individuals responsible for the aggression. Child C4 does not respond. W5 asks the same question to the group. Some children say “No”, but others suggest “apologizing” or “giving a hug”*.

“*What can we do to avoid these situations?” W5 asks. “Not hurting others,” a child proposes*.

“*Do you all agree?” W5 asks*.

“*Yes,” they respond unanimously. W5 proposes to the group that they think of a consequence whenever the rule is not followed. Several suggestions are made: apologizing, helping those who have been hurt (Field note, 10/11/2022)*.

The systematic and persistent guidance provided by adults in regulating relationships among children, but above all, the opportunity to break away from a certain naturalization of a pattern characterized by a lack of conviviality among children and a lack of space for each child's participation, seemed to us a fundamental strategy in negotiating their rights and responsibilities. It represented a significant moment in promoting the co-creation dimension of education in common, demonstrating their agency and responsibility in shaping their own community.

Thus, the children faced the scenario that they had to define for themselves, and they had the possibility to consider that the reality could be different, that there could be room for change. This contributed to further naturalized patterns in that context and space, which sometimes leaned toward punitive measures and conviviality and disrespect for others' rights.

**Figure 2:**
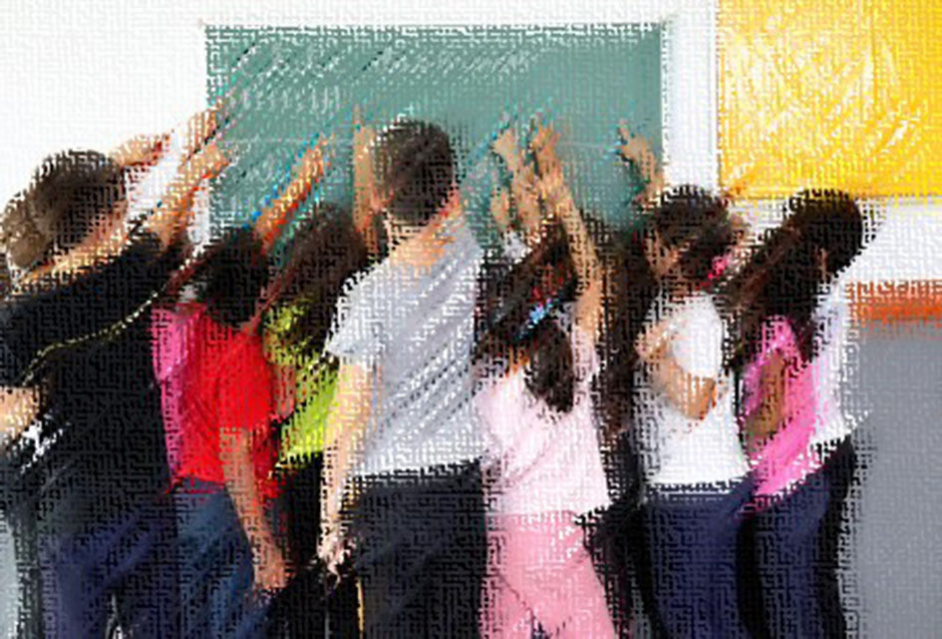
Collective decision-making process to select the name of the space.

Another noteworthy episode was the process of collective decision-making used to democratically select a name for the space used by the children during the project (see Figure 2).

The following field note provides details on how this process unfolded:

*The adult suggested dividing the children into small groups to work on an activity related to choosing a name for the space to be used by the children. Although the activity was accompanied by a high level of noise and some initial reluctance from the children to participate, they started discussing possible names for the space. After decision-making in small groups, in a later phase, the proposals were shared with the larger group, leading to further discussion and decision-making, which resulted in the final name for the space (Field note, 23/06/2022)*.

Based on the image of children as capable social actors, as discussed earlier, their participation takes on specificities that have been highlighted by various authors in support of a broader political approach. In other words, participation that truly empowers children and brings improvements to their well-being, as well as promoting their social inclusion, as emphasized by Pechtelidis and Kioupkiolis (2020):

“*Participation is closely associated with children's agency. The notion of children's participation in the social process of common goods gives a practical and political dimension to the idea of agency, emphasizing children's capabilities and their shaping influence in their environments” (Oswell*, *2013**; Valentine*, *2011**; Wyness*, *2013*, *2018**, p. 2)*.

Conviviality, understood as a social practice based on cooperation, solidarity and dialogue, is a fundamental element for the construction of common spaces. This ability for individuals to coexist in harmony, respecting each other and sharing spaces, values, and experiences was a central dimension fostered among the children of the Children's Club.

In the Children's Club, according to the analysis of the dynamics, it was frequent for the children not to prioritize the construction of community relationships, with each one asserting their own interests and schedules, revealing many difficulties in organizing themselves as a collective. However, on certain occasions, the children were able to interact harmoniously, as in the case of a chair game activity, where they were able to complete the game without significant conflict. During free playtime, there were episodes of physical aggression (including hitting, punching, kicking, pushing, throwing objects, or any other physical act intended to cause harm or injury), usually involving the same children, both boys and girls, with a greater predominance among boys.

Another observation concerns aggressive and violent behavior, especially among boys, as a way to deal with conflicts. This may indicate that these children need to develop social skills to handle conflict situations without resorting to violence. However, it is essential to prioritize listening and respect for others during this process of interaction. It was also observed that collaboration is evident in certain activities throughout the sessions, such as playing the drum or cleaning the room at the end of the activities, meaning they actively engage in activities they enjoy (Field note, 23/06/2022).

In general, it seems that the children's ability to prioritize community relationships over individual interests depended on the specific context and situation they were in, as well as their individual personalities and preferences, and the systematic and persistent work of the educators from Porta 7 and the Smooth team in this regard. The following testimony is indicative of this situation:

“*It has improved, and I'll explain why. Because before, because I, I, within myself, even at home, my mind was already very stressed. I am already very stressed. And in the children's club, my mind has become a little more patient” (Child C6)*.

The children demonstrate a constant willingness to speak freely, without worrying if others are listening or if they are hindering others' ability to hear, which often presents itself as a communication problem for the group and the difficulties in internalizing conviviality rules that allow everyone to exercise their right to expression.

## Discussion

As discussed, education as a common good requires ongoing reflection on what “good” and “common” mean, as well as an understanding of human values and the role of education in promoting those values in society.

This article focused on the importance of children's participation in the collective construction of the common good, emphasizing the reflection on the child as a commoner. Children's participation in processes related to their lives was a fundamental dimension in the intervention with children.

The “Children's Club” allowed for the creation of a physical and symbolic space for shared governance meaningful for children and adults. Regarding common practices among children and between children and adults, the importance of using pedagogies of cooperation and solidarity as the basis for a new “education contract” (UNESCO, 2021) was emphasized. Through observations, several situations were identified that demonstrated differentiated attitudes toward sharing and caring. It was found that the children in the group had significant difficulty in sharing and caring for their peers, but they showed caring attitudes toward their close ones (family and friends). These practices help children develop essential social and emotional skills, creating an inclusive and supportive environment where inequalities are addressed, and a sense of belonging is nurtured.

Regarding cooperation and collective creativity in education, their importance and how these dimensions gradually developed in the Children's Club project were discussed. The main activity that illustrated these dimensions was the composition of a song, which involved sharing and discussing ideas in small groups, culminating in the creation of an anthem. Working in small groups proved to be strategic for children to listen to their peers' ideas and participate in decision-making regarding the song. Therefore, it can be stated that these activities promoted collaboration, shared responsibility, and mutual respect within this group of children. Cooperation and collective creativity within the Children's Club enhance the process of reversing inequalities by fostering collaboration, creating renewed social practices, promoting active participation and creativity, cultivating collaboration and decision-making, and demonstrating a sense of community identity. These practices empower children, encourage their active involvement, and contribute to the development of a supportive and inclusive environment that addresses and challenges inequalities.

The idea that participation and active citizenship are fundamental in educational processes that promote the common good was present throughout the project, aiming to overcome the obstacles experienced by children in a community where they are often seen as incapable of participating in public life (Cappello and Siino, 2023). Thus, in order to achieve full active citizenship, educational institutions must create spaces for children's autonomous expression, providing them with opportunities to be reflective, autonomous, and socially active, as was the case with the Children's Club. These practices empower children, encourage their active engagement, and contribute to the development of an inclusive and equitable environment that values their voices, agency, and contributions. Additionally, the dimension of cooperation is highlighted when children and adults come together in the Club's space, emphasizing the dimension of participation and sharing when the space allows access to information and supports problem-solving, ultimately promoting social inclusion.

Also, involving children in defining rules and responsibilities within the group promotes collective negotiations and collaborative efforts to achieve a healthy environment for coexistence. Regarding the dimension of conviviality, perceived here as a social practice based on cooperation, solidarity, and dialogue, it proves to be essential for the construction of common spaces. As observed, children's ability to prioritize community relationships over individual interests depends on the context and specific situation they are in. Faced with difficulties in managing their negative emotions appropriately, children use strategies such as speaking out of turn, leading to conflicts. This demonstrates the urgent need to promote individual and social skills that facilitate coexistence based on mutual respect. Nevertheless, concerning the conviviality dimension, it became evident that involving children in defining rules and responsibilities within the group is important, promoting collective negotiations and collaborative efforts to establish a healthy environment that enhances the process of reversing inequalities by recognizing all children as equal active participants.

In this research, the main challenges in building a common good space in non-formal education resided in establishing important assumptions of educational action, such as sharing and caring to foster cooperation and solidarity among children and between children and adults, as well as achieving shared responsibility and mutual respect. By fostering these practices and values, educational institutions can create environments where children are seen as capable social actors and actively contribute to shaping a more equitable and inclusive society. Without safeguarding these aspects, it becomes challenging to develop participation dynamics and active citizenship, which are fundamental dimensions in educational processes that aim to promote the common good and operate as catalysts for reversing inequalities.

## Data availability statement

The datasets presented in this article are not readily available because of project ethical procedures. Requests to access the datasets should be directed to NF, natfs@ie.uminho.pt.

## Ethics statement

The studies involving human participants were reviewed and approved by the University of Minho Ethics Committee (CEICSH 055/2021). Written informed consent to participate in this study was provided by the participants' legal guardian/next of kin.

## Author contributions

All authors listed have made a substantial, direct, and intellectual contribution to the work and approved it for publication.
